# Functional Exploration of the Adult Ovarian Granulosa Cell Tumor-Associated Somatic *FOXL2* Mutation p.Cys134Trp (c.402C>G)

**DOI:** 10.1371/journal.pone.0008789

**Published:** 2010-01-20

**Authors:** Bérénice A. Benayoun, Sandrine Caburet, Aurélie Dipietromaria, Adrien Georges, Barbara D'Haene, P. J. Eswari Pandaranayaka, David L'Hôte, Anne-Laure Todeschini, Sankaran Krishnaswamy, Marc Fellous, Elfride De Baere, Reiner A. Veitia

**Affiliations:** 1 Programme de Pathologie Moléculaire et Cellulaire, Institut Jacques Monod, Paris, France; 2 Université Paris Diderot/Paris 7, Paris, France; 3 Ecole Normale Supérieure de Paris, Paris, France; 4 Center for Medical Genetics, Ghent University Hospital, Ghent, Belgium; 5 School of Biotechnology, Kamaraj University, Madurai, India; 6 Département de Génétique et Développement, Institut Cochin, Paris, France; Cincinnati Children's Research Foundation, United States of America

## Abstract

**Background:**

The somatic mutation in the *FOXL2* gene c.402C>G (p.Cys134Trp) has recently been identified in the vast majority of adult ovarian granulosa cell tumors (OGCTs) studied. In addition, this mutation seems to be specific to adult OGCTs and is likely to be a driver of malignant transformation. However, its pathogenic mechanisms remain elusive.

**Methodology/Principal Findings:**

We have sequenced the *FOXL2* open reading frame in a panel of tumor cell lines (NCI-60, colorectal carcinoma cell lines, JEG-3, and KGN cells). We found the *FOXL2* c.402C>G mutation in the adult OGCT-derived KGN cell line. All other cell lines analyzed were negative for the mutation. In order to gain insights into the pathogenic mechanism of the p.Cys134Trp mutation, the subcellular localization and mobility of the mutant protein were studied and found to be no different from those of the wild type (WT). Furthermore, its transactivation ability was in most cases similar to that of the WT protein, including in conditions of oxidative stress. A notable exception was an artificial promoter known to be coregulated by *FOXL2* and Smad3, suggesting a potential modification of their interaction. We generated a 3D structural model of the p.Cys134Trp variant and our analysis suggests that homodimer formation might also be disturbed by the mutation.

**Conclusions/Significance:**

Here, we confirm the specificity of the *FOXL2* c.402C>G mutation in adult OGCTs and begin the exploration of its molecular significance. This is the first study demonstrating that the p.Cys134Trp mutant does not have a strong impact on *FOXL2* localization, solubility, and transactivation abilities on a panel of proven target promoters, behaving neither as a dominant-negative nor as a loss-of-function mutation. Further studies are required to understand the specific molecular effects of this outstanding *FOXL2* mutation.

## Introduction

*FOXL2* encodes a protein belonging to the Forkhead/Winged-Helix family of transcription factors. In 2001, its mutations were implicated as the cause of the Blepharophimosis Ptosis Epicanthus-inversus Syndrome (BPES; MIM 110100) in humans [1]. This genetic disorder is characterized by craniofacial abnormalities, either isolated (BPES type II) or in association with premature ovarian failure (BPES type I) [2]. FOXL2 has been detected in the nuclei of ovarian granulosa cells, pituitary thyrotrope and gonadotrope cells and in the peri-ocular mesenchyma [3], [4], [5], [6] but some lines of evidence suggest the existence of a wider expression pattern [7], [8], [9]. Numerous germline FOXL2 mutations have been described in association with BPES, including missense, nonsense, frameshift mutations and in-frame expansions of the polyalanine tract [10].

Recently, two clinical studies have linked somatic perturbations of FOXL2 with the occurrence of Ovarian Granulosa Cell Tumors (OGCT) [11], [12]. OGCTs are a rare form of malignancy affecting women of all ages, with two distinct clinical presentations for the adult and juvenile forms [13]. Most juvenile cases are diagnosed early and their prognosis is generally good, though recurrences and metastases have been reported [14]. However, in the adult cases of OGCTs, 20% of patients die of the consequences of their tumor, with a 5-year survival of advanced oncological stage patients being less than 50% [13]. These tumors have a tendency to late recurrence, with latency after primary tumor treatment of up to 37 years. Moreover, chemotherapy has limited success and surgery remains the main treatment option.

In an effort to identify a specific marker of adult OGCTs, Shah and collaborators uncovered a single recurrent somatic *FOXL2* mutation (c.402C>G; p.Cys134Trp, hereafter referred to as C134W) through high-throughput whole-transcriptome sequencing of four independent OGCT samples. Moreover, they confirmed it in 86 out of 89 independent OCGT samples [12]. Interestingly, Cys134 is a highly evolutionarily conserved residue in the FOXL2 protein sequence, which is predicted to lie in the second Wing structure of its Forkhead/Winged-helix domain.

Interestingly, FOXL2 has been suggested to be a potential tumor suppressor in colorectal cancer (CRC) [15]. Indeed, the *FOXL2* locus was found to be hypermethylated in CRC cell lines and primary tumor samples with inhibition of its expression, whereas the locus was unmethylated in healthy tissue. Moreover, FOXL2 overexpression inhibited colony formation in the HCT116 CRC cell line (although the contribution of some degree of toxicity to this phenomenon remains to be determined). Thus, FOXL2 might act as a tumor suppressor in a more systematic way than anticipated. We have reviewed in detail other molecular links between FOXL2 and tumor suppression according to the known literature, and proposed that FOXL2 could indeed act as a novel tumor suppressor [8].

In this study, we determined the sequence of *FOXL2* in a panel of tumor cell lines (NCI-60 panel, 34 established CRC cell lines, as well as the JEG-3 and KGN cell line). In order to better understand the pathogenic mechanism of the C134W mutation, we set out to characterize its molecular behavior. Specifically, we studied the subcellular localization of the FOXL2-C134W variant, as well as its mobility. We analyzed its transactivation ability, as compared to known type I or II BPES associated variants, on known targets, in two different cell lines. Finally, we also generated a 3D-structural model of the C134W variant dimer to compare with the WT one.

## Results and Discussion

### *FOXL2* Genotyping in Established Tumor Cell Lines of Various Tissue Origins

In order to assess the potential implication of the somatic mutation c.402C>G in the malignant transformation of various tissues including the ovary, we sequenced *FOXL2* in a large variety of established and well-characterized cancer cell lines. For this purpose, we obtained genomic DNA from 51 different cancer cell lines from the NCI-60 panel. We also sequenced the genomic DNA from the adult OGCT cell line KGN, to uncover whether it carried the described recurrent mutation c.402C>G [16], as well as the choriocarcinoma cell line JEG-3, derived from placental tissue, in which mRNA *FOXL2* expression has been detected (GEO dataset GSE13475; [17]). Moreover, as the methylation status of the *FOXL2* locus was found to be modified in colorectal cancers (CRC)[15], we hypothesized that other inactivating mechanisms, such as somatic mutations in the coding region, might also be involved in CRC pathogenesis. Therefore, we also analyzed the *FOXL2* genomic DNA sequence from 38 CRC cell lines [18].

Briefly, the c.402C>G (p.Cys134Trp) somatic mutation was found at the heterozygous state in the KGN cell line (Figure S1), but was not detected in any other cell line (Table S1). We also found several occurrences of the known polymorphisms [c.501C>T (p.Phe167Phe), dbSNP: rs61750361 and c.536C>G (p.Ala179Gly), dbSNP: rs7432551] that were systematically found to occur in *cis*, whether at the homozygous (hemizygous?) or heterozygous state (Figure S1). A new silent polymorphism was also detected in the NCI-H460 Non-Small Cell Lung cancer cell line as well as in the colorectal cancer cell line HCA7 (c.858T>G, i.e. p.Pro257Pro; Figure S1). Consistently, while this paper was in preparation, Schrader and collaborators published the absence of the c.402C>G mutation in the NCI-60 cell line panel and its presence at the heterozygous state in KGN cells, underlying its specificity for OGCTs [19].

The absence of the c.402C>G mutation in other types of cancer than adult OGCT, such as in CRC, does not necessarily negate a potential implication of FOXL2 inactivation in the pathogenesis process. An epigenetic mechanism underlying *FOXL2* inactivation cannot be excluded, as somatic hypermethylation of the *FOXL2* locus was recently demonstrated in CRC [15]. From an epigenetic viewpoint, it is also noteworthy that inactivation of FOXL2 in juvenile OGCT cases is correlated with tumor mitotic index and aggressivity, although *FOXL2* mutations are rarely present [11], [12].

Apart from epimutations, the occurrence of regulatory variants within the untranslated regions (UTRs), the core promoter or long-range regulatory elements should be considered. Indeed, deletions involving long-range regulatory regions containing Conserved Non-Genic sequences (CNGs) have recently been reported at the constitutional level as a cause of BPES [9], [20]. In addition, a constitutional variation in the *FOXL2* 3′UTR was found to cosegregate with BPES in a large Chinese pedigree [21], and might disturb *FOXL2* mRNA stability. Briefly, it would be possible that a perturbation of *FOXL2* expression through somatic hypermethylation, locus heterozygous deletion or regulatory mutations (i.e. in enhancer sequences, promoter, UTRs,…) could play a role in malignant transformation, in a context where no mutations in the coding region are detected. However, further insights into the effects of the C134W mutation are required to assess the plausibility of these hypotheses.

The Cys134 residue is conserved among a few forkhead paralogs, including the only other member of the L subfamily, FOXL1. Interestingly, FOXL1 has been shown to play a role in carcinogenesis [22]. We therefore sequenced *FOXL1* in the NCI-60 cell line panel. We failed to detect any alteration of codon 129, encoding the Cys paralogous to Cys134 in FOXL2. However, we found several known or new polymorphisms in *FOXL1*, which are reported in the Table S2.

### Subcellular Localization and Mobility of the FOXL2-C134W Protein

Most of the described FOXL2 BPES-associated mutations have been found to induce either nuclear aggregation or cytoplasmic delocalization and aggregation, and this has been suggested to participate in the BPES pathogenesis at the molecular/cellular level [23], [24], [25], [26], [27]. Interestingly, missense mutations in the Forkhead domain of FOXL2 often lead to subcellular mislocalization and protein aggregation [23]. Moreover, when investigated, FOXL2 protein aggregation was associated with impaired FOXL2 protein mobility [25]. Therefore, we studied the subcellular localization of a FOXL2-C134W-GFP fusion protein in COS-7 cells, which are readily transfectable and that we have often used to study mutant FOXL2 localization. We observed no significant aggregation or mislocalization of the fluorescently-tagged protein (data not shown). In this respect, the mutant protein behaves quite similarly to the wild-type (WT) one. Our observations in transfected cells are compatible with the immunohistochemistry findings by Shah and collaborators in tumors carrying the mutation, which suggested no effect on protein localization [12].

**Figure 1:**
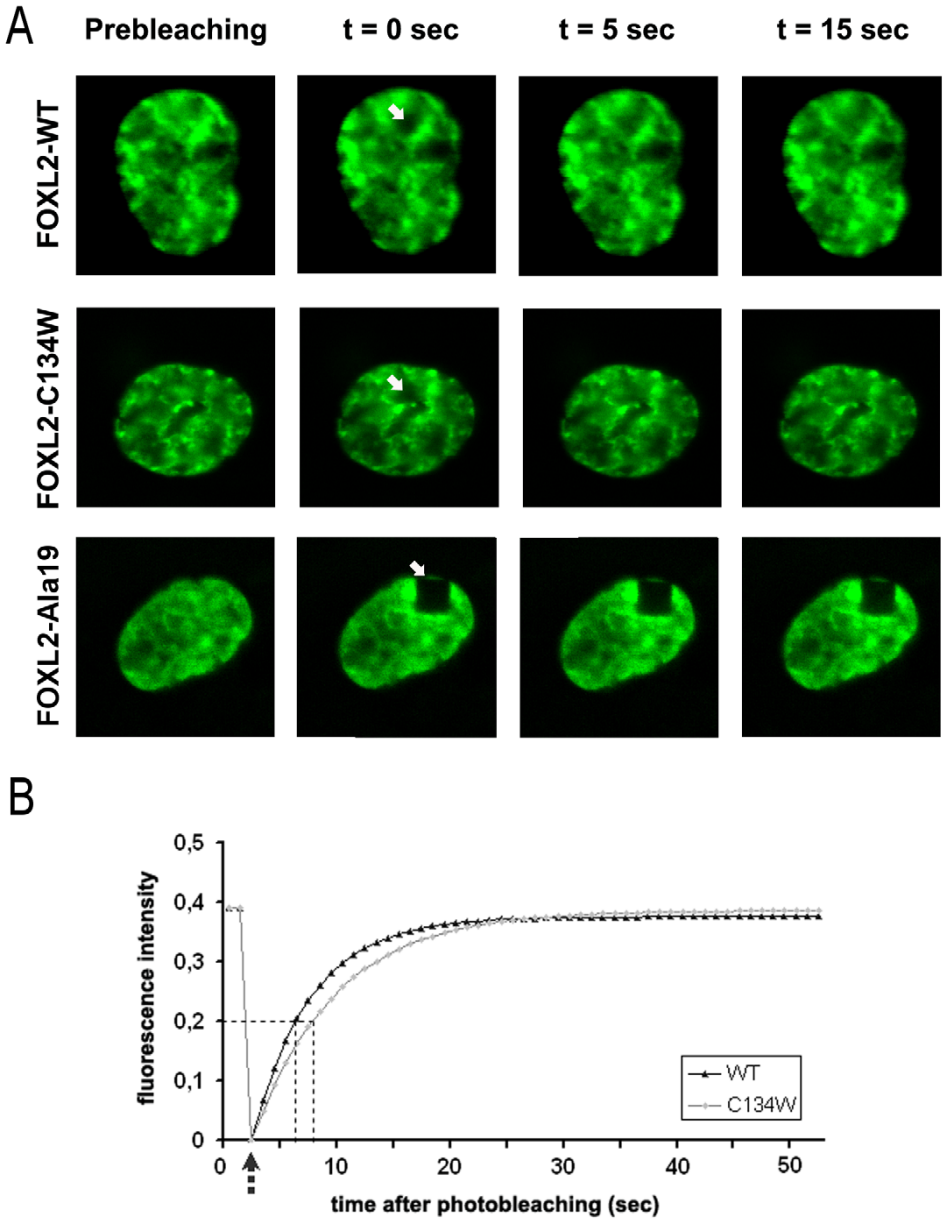
Analysis of FOXL2-C134W-GFP protein mobility by Fluorescence Recovery after Photobleaching in COS-7 cells nuclei. (A) In each mutant case, the leftmost panel shows the GFP fusion proteins in the nuclei prior to photobleaching, the second panel (0 s) shows the GFP fusion proteins immediately after bleaching (the white arrows indicate the bleached portion, which appear as black regions). The next two panels show fluorescence recovery after 5 and 15 s respectively. FOXL2-Ala19-GFP is shown as a control FOXL2 mutant which has a rather homogeneous nuclear distribution in transfected cells, but whose protein mobility is compromised, as described previously [25]. Notice that Fluorescence is recovered for both the WT and C134W variants, but not the Ala19 variant. (B) Graphs reporting fluorescence in the bleached zone as a function of time are reported. Data was computed for 21 cells and 22 cells for the WT and C134W FOXL2 variants respectively, and the average fluorescence values are plotted. Fluorescence was recovered in both cases, and recovery times were not significantly different between the two different FOXL2-GFP variants.

However, as at least one mutant FOXL2 protein presents an impaired protein mobility without visible aggregation (namely, the pathogenic expansion of the polyalanine domain to 19 residues; [25]), we investigated whether the mobility of the FOXL2-C134W was different from that of the WT protein, also including as a control the FOXL2-Ala19 variant (Figure 1). The mutant protein did not display any perturbation of its mobility as compared to the WT (t1/2 = 7.06±0.81 and t1/2 = 6.05±1.08 respectively; p = 0.45), contrary to the FOXL2-Ala19 variant, which was found unable to recover from photobleaching, as previously described [25]. This suggests that FOXL2-C134W is fully diffusible in the nucleus, and that it should be able to readily reach its target promoters.

### Transactivation Capacity of FOXL2-C134W on Known Target Promoters

**Figure 2:**
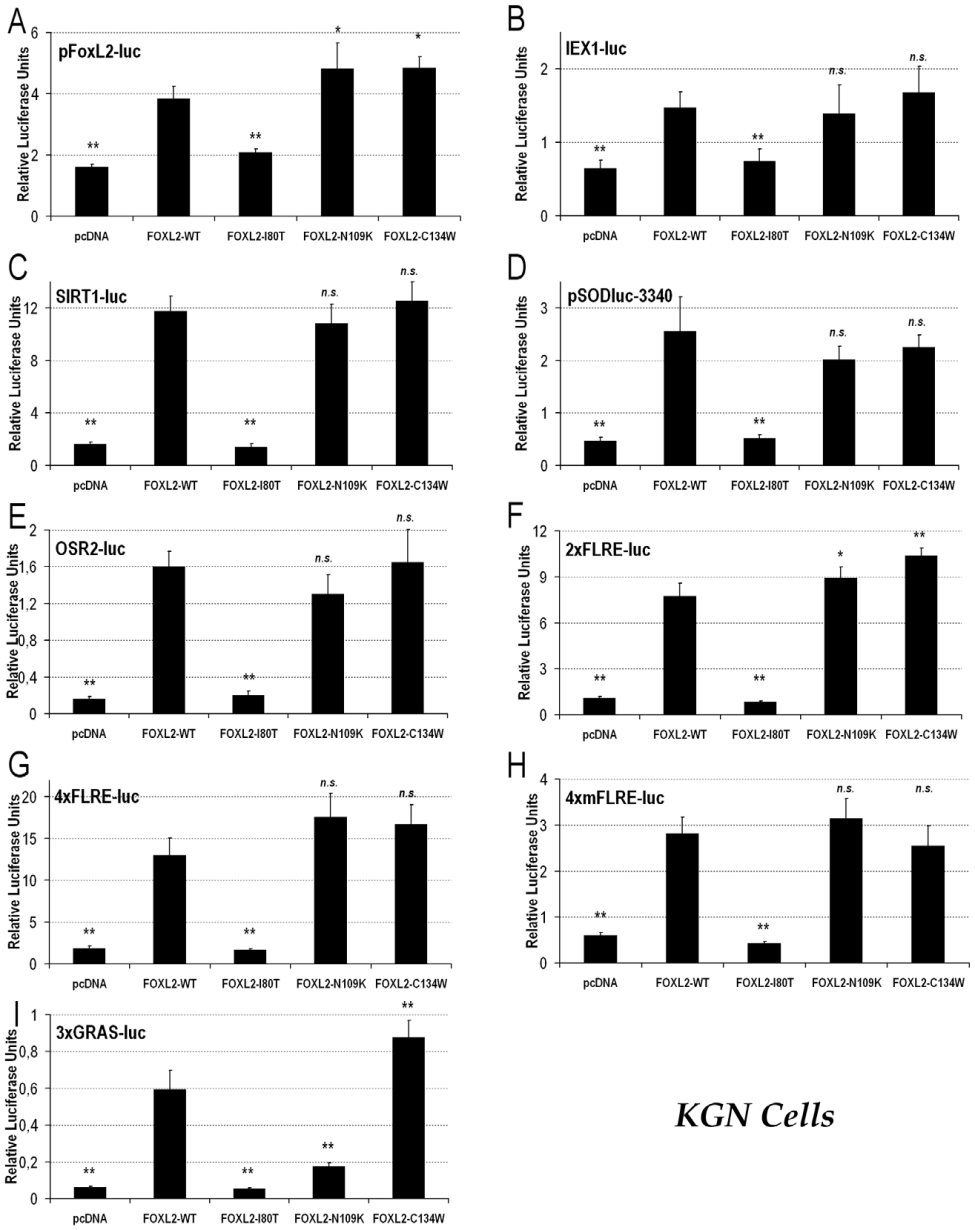
Luciferase assays show that the FOXL2-C134W variant seems fully functional in adult OGCT KGN cells. Luciferase assays in KGN cells transfected with pFoxL2-luc (A), IEX1-luc (B), SIRT1-luc (C), pSODluc-3340 (D), OSR2-luc (E), 2xFLRE-luc (F), 4xFLRE-luc (G), 4xmFLRE-luc (H) or 3xGRAS-luc (I). In each case, the experiment is conducted in presence of either the empty control vector (pcDNA3.1-GFP), or the expression vectors for the FOXL2-WT version, the representative BPES type I-inducing variant FOXL2-I80T, the representative BPES type II-inducing variant FOXL2-N109K or the OCGT-associated FOXL2-C134W version. Statistical significances from an ANOVA, followed by post-hoc Tukey HSD tests, for values compared to FOXL2-WT are reported. n.s.: non-significant; *: p<0.05; **: p<0.01.

Since subcellular localization and solubility of FOXL2-C134W were not significantly different from those of the WT protein, then we studied its transactivation capacity on a panoply of luciferase reporters known to respond to FOXL2, looking for potential dysregulations. We either used reporters under the control of naturally-occurring promoter sequences (pFoxL2-luc, IEX1-luc, SIRT1-luc, pSODluc-3340 and OSR2-luc) or under the control of artificial promoters responsive to FOXL2 (2xFLRE-luc, 4xFLRE-luc, 4xmFLRE-luc and 3xGRAS-luc). Using these reporters to assess the molecular phenotype of the FOXL2-C134W mutant is relevant, because their promoters have been previously shown to display various affinity levels and specific behaviors towards distinct types of FOXL2 mutations [23], [25], [27], [28]. As control mutations, we also included a representative BPES type I-causing variant (FOXL2-I80T), which is associated with granulosa cell dysfunction, and a representative BPES type II-causing variant (FOXL2-N109K), which is associated with normal ovarian function, to compare the molecular behavior of the FOXL2-C134W cancer-associated variant with BPES-causing mutations. First, we performed the luciferase assays in the adult OGCT-derived KGN cell line, even if it carries the p.Cys134Trp mutation at the heterozygous state, because these cells have been shown to be good models of granulosa cells [16] and because, we hypothesized, overexpression of the FOXL2 variants should outweight the endogenous protein levels in this kind of assay (Figure 2).

**Figure 3:**
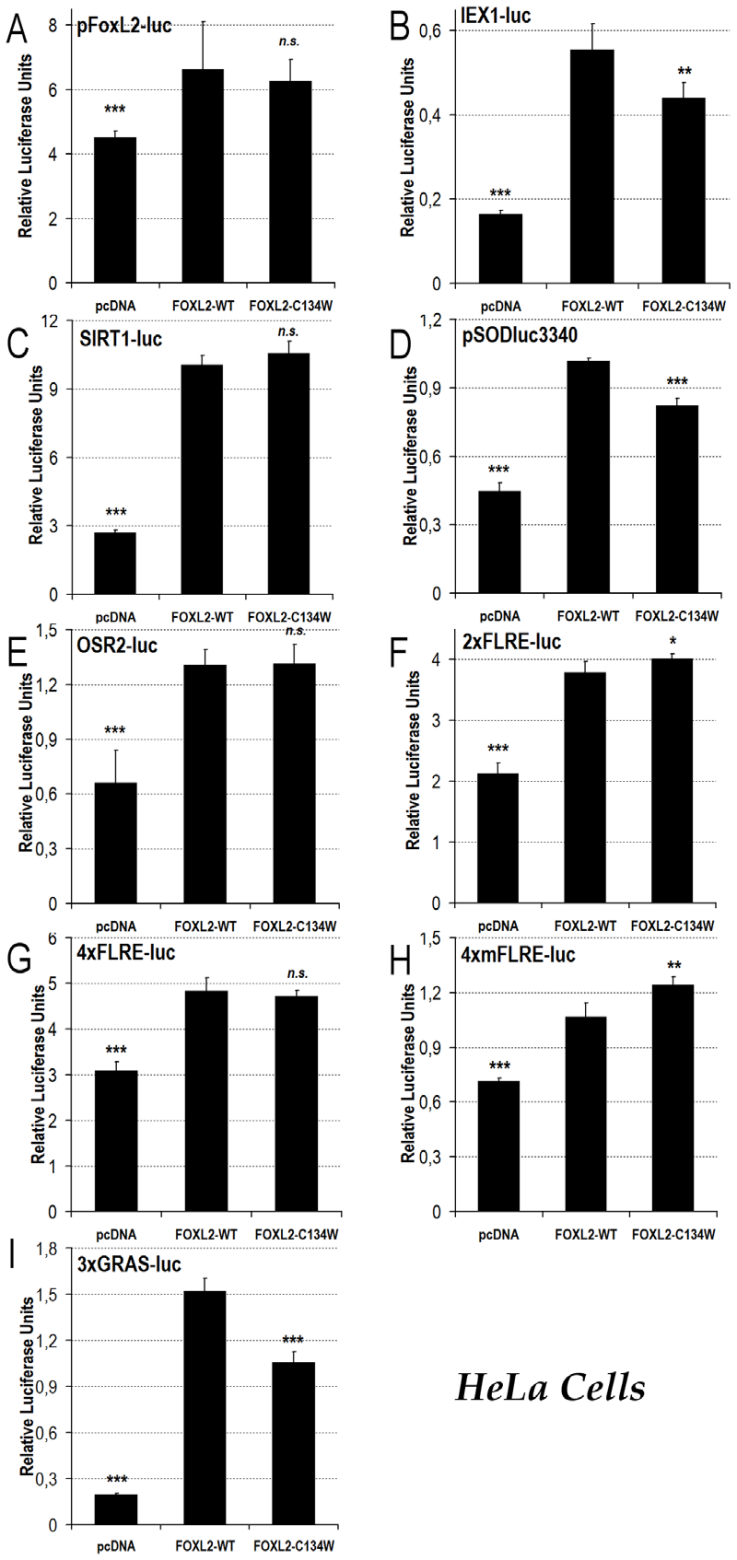
Luciferase assays show that the FOXL2-C134W variant seems functional in cervical cancer HeLa cells. Luciferase assays in HeLa cells transfected with pFoxL2-luc (A), IEX1-luc (B), SIRT1-luc (C), pSODluc-3340 (D), OSR2-luc (E), 2xFLRE-luc (F), 4xFLRE-luc (G), 4xmFLRE-luc (H) or 3xGRAS-luc (I). In each case, the experiment is conducted in presence of either the empty control vector (pcDNA3.1-GFP), the expression vector for the FOXL2-WT version, or the OCGT-associated FOXL2-C134W version. Statistical significances from an ANOVA, followed by post-hoc Tukey HSD tests, for values compared to FOXL2-WT are reported. n.s.: non-significant; *: p<0.05; **: p<0.01; ***p<0.001.

In most cases, FOXL2-C134W behaved similarly to the WT protein (Figure 2B–E, 3G–H), as is the case for the typical type II-associated missense mutant FOXL2-N109K, whereas the type I-associated mutant FOXL2-I80T always behaved like a null mutation. In this respect, the behavior of type I and II-associated variants is consistent with previous studies [27]. In other instances (A, F), both FOXL2-C134W and FOXL2-N109K present an activity slightly enhanced as compared to the wild-type protein. These increases are not necessarily relevant from a physiological viewpoint, as BPES type II-associated mutants, such as FOXL2-N109K, do not impair granulosa cell function [1], [2], [27], and induce no ovarian cancer predisposition, as in all cases of BPES reported so far.

Surprisingly, in the particular case of 3xGRAS-luc, the FOXL2-C134W variant seemed hyperactive as compared to the WT protein (I), whereas both FOXL2-I80T and FOXL2-N109K were hypomorphic. It is noteworthy that this is the first published occurrence of a BPES type II-associated missense variant displaying such low activity on a promoter reporter, which strongly suggests that the 3xGRAS-luc reporter is a very low-affinity target of FOXL2. Reporter 3xGRAS-luc (GRAS: GnRH Receptor Activating Sequence) is a synthetic reporter, containing three repeats of a composite regulating element (overlapping AP1, Smad3 and Foxl2 binding sites) which is found in the murine promoter of the *GnRH Receptor* (*GnRHR*) and seems to contribute to the regulation of its expression in pituitary gonadotrope cells [29]. It has been shown that this sequence is bound by a Smad3-Foxl2 heterodimer [29]. Though it is best known and studied in the pituitary, recent evidence suggests that the GnRH/GnRHR system is present in the ovary [30], [31]. However, the physiological implications of this are still unclear. Our luciferase results therefore seem to indicate that the p.Cys134Trp mutation could induce a disruption of normal FOXL2/SMAD3 interaction.

To address the concern that half of endogenous FOXL2 in KGN cells involves the very C134W variant, which is under study, we decided to study the effects of the p.Cys134Trp mutation in the cervical cancer HeLa cell line (Figure 3). According to the BioGPS portal, HeLa cells (from the NCI-60 panel) express *FOXL2* at detectable levels (http://biogps.gnf.org/#goto=genereport&id=668; U133A gcrma, chip 220102_at), so they should be able to manage transfected FOXL2 and add the correct post-translational modifications necessary for FOXL2 activity. Moreover, according to *FOXL2* EST expression profile from the Unigene database (Hs.289292), *FOXL2* is expressed at significant levels in both healthy uterus and cervix, which makes HeLa cells, as a cervical cell line, a fairly good model to study FOXL2 activity.

In the case of the pFoxL2-luc, SIRT1-luc, OSR2-luc, 2xFLRE-luc and 4xFLRE-luc reporters, the behavior of the FOXL2-C134W mutant was much like what we had observed in KGN cells (Figure 3A, C, E–G). Interestingly, on the target stress-response reporters promoter (i.e. MnSOD = pSODluc3340 and IEX1/IER3 = IEX1-luc), we found that the FOXL2-C134W mutant was just slightly, but significantly, hypomorphic in HeLa cells, whereas it had similar activity to the WT in KGN cells (Figure 3B, D). In our opinion, this could indicate that according to different proteomic contexts (i.e. HeLa versus KGN), the FOXL2-C134W mutant may lead to slightly different (impaired?) stress responses. We also found that FOXL2-C134W was slightly hypermorphic on the 4xmFLRE-luc promoter in HeLa cells, whereas it showed WT-like activity in KGN cells (Figure 3H). However, the biological relevance of this finding is to be determined since the 4xmFLRE-luc reporter is an artificial one.

Finally, on the 3xGRAS-luc reporter, the FOXL2-C134W mutant protein was hypomorphic as compared to the WT in HeLa cells, which would indicate once again a potentially disrupted FOXL2/SMAD3 interaction (Figure 3I). This result seems to contradict what we obtained in KGN cells, where FOXL2-C134W was hypermorphic on this articifial promoter. However, as KGN and HeLa cells are derived from different tissues, we cannot exclude cell-type-dependent differences of SMAD heterodimerization with FOXL2. Moreover, as 3xGRAS-luc is an artifical promoter, and that other transcription factor binding sites surrounding the sites in naturally occurring promoters are lacking, fine tissue-dependent regulations can be absent and might induce such an apparently contradictory behavior.

It has recently been shown that mouse Foxl2 interacts with Smad3 through the C-terminal part of its Forkhead domain [32]. The Forkhead domain sequence of human FOXL2 and mouse Foxl2 are strictly conserved [4], and the Cys134 residue of the human sequence is homologous to Cys130 in the mouse sequence of FOXL2. The sequence encompassing amino acids 132 to 161 in the mouse Foxl2 protein sequence was found to be crucial for Foxl2/Smad3 interaction [32]. The proximity between the interaction motif of Foxl2 and Smad3 and the recurrent Cysteine residue mutated in cancer, in conjunction with the atypical behavior of FOXL2-C134W on the known Foxl2/Smad3 target 3xGRAS-luc in both KGN and HeLa cells, may suggest that the specific pathogenesis of this cancer-related mutation could result, at least in part, from a modification of the Foxl2-Smad interaction in the regulation of a subset of ovarian targets genes. This is particularly relevant in the case of a cancer-associated mutation, since Smad3 has been shown to be a key mediator of the TGF-β cytostatic program [33]. All in all, these findings and hypotheses require further studies in the context of naturally-occurring ovarian FOXL2-responsive promoters containing FOX-SMAD binding sites. Such promoters are, unfortunately, yet to be characterized.

**Figure 4:**
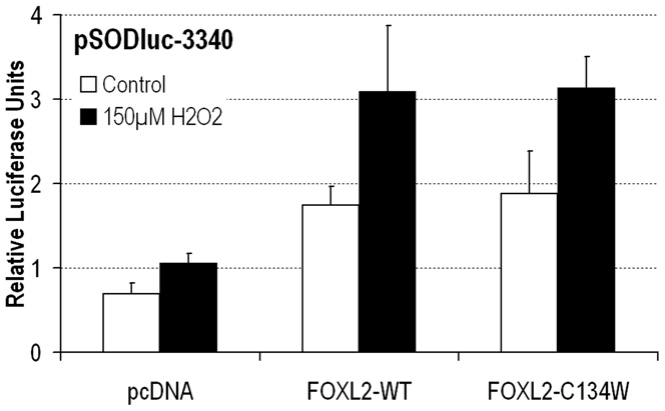
FOXL2-C134W transactivation is increased upon oxidative stress to the same extent as for the WT version. Luciferase assays in KGN cells using the MnSOD promoter reporter pSODluc-3340, with empty control vector, FOXL2-WT or FOXL2-C134W overexpression vectors, to assess the effect of a 2h-treatment with 150 µM H_2_O_2_ on the activity of the FOXL2 versions. The increase of FOXL2 transactivation ability observed upon oxidative stress, which we have previously reported for WT FOXL2 [34], is similar (i.e. not statistically different) in the cases of the WT and of the cancer-associated FOXL2-C134W variant.

We have recently shown that FOXL2 is involved in the cellular response to oxidative stress [34]. Among other things, we have shown that FOXL2 transactivation was increased in response to cell stress, notably on the promoter of the mitochondrial Manganese Superoxide Dismutase (MnSOD), where the increase in transactivation was stress-dose dependent. This was suggested to help ovarian granulosa cells deal with a stress event [34], [35]. Furthermore, an altered response to oxidative stress can have genotoxic consequences, which is particularly important in the ovary, considering the massive reactive oxygen species production during ovulation [36]. Therefore, we studied whether the FOXL2-C134W mutant protein was able to properly respond to an oxidative stress stimulus using a stress-response reporter driven by the *MnSOD* promoter (pSODluc-3340, Figure 4).

Oxidative stress of KGN cells induced an increase of transactivation of both the WT and the C134W mutant to a similar extent, suggesting that the FOXL2-C134W variant is able to respond appropriately to stress. Therefore, the potential defect induced by the p.Cys134Trp mutation might not lie in an altered response to cellular stress (which can contribute to the appearance and development of cancer). However, further studies may be necessary to completely rule out a potentially dysregulated stress response in the pathogenesis of the p.Cys134Trp mutation.

### 3D-Modeling of the Consequences of FOXL2 Mutation p.Cys134Trp

**Figure 5:**
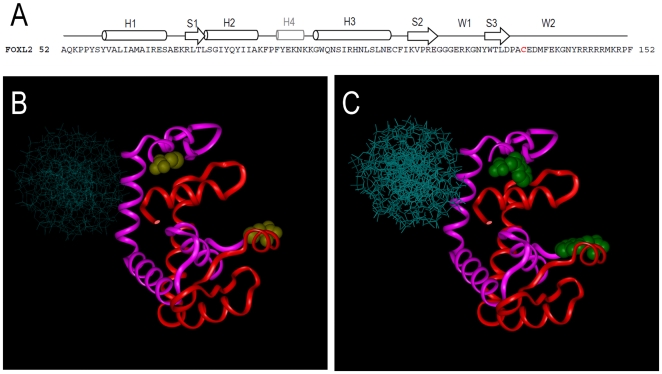
3D structural model of WT and C134W FOXL2 proteins. (A) FOXL2 Forkhead domain protein sequence. Secondary structures are indicated at the top, with barrels for α-helices (H1 to H4) and open arrows for β-strands (S1 to S3). Black symbols indicate structures typical of all Forkhead domains, while H4 is indicated in gray, as it was predicted to exist in the case of FOXL2 by sequence homology, though no crystal data is directly available. Cys134 is highlighted in red in the predicted second Wing structure. (B–C) Structural models of the FOXL2 forkhead domains in a dimer conformation, fitted to the experimental structure of the FOXP2/DNA crystal [37]. WT (B) and C134W (C) dimers are represented. The proteins are displayed as red and pinks ribbons and DNA is shown in blue as a stick model. The residue at the 134^th^ position is shown as a molecular model in 3D.

The forkhead domain of FOXL2 can be structurally accommodated into the same tertiary and quaternary association which was reported for the forkhead domain of FOXP2 [37]. We have previously obtained a preliminary model in order to explore the possible effects of FOXL2 disease-causing mutants according to their predicted structural positioning [26]. We had then shown that it was possible to sort the mutants into two classes: those that should alter protein interactions within and between FOXL2 molecules and those that should disrupt the interaction of FOXL2 with DNA [26]. The 3D-models for WT-WT and C134W-C134W protein homodimers bound to DNA are displayed in Figure 5.

Our model predicts that the cancer-associated p.Cys134Trp mutation is likely to affect the protein/protein homotypic interaction. Unfortunately, the protein region beyond the forkhead is not present in our model, since there is no readily available structural template, so potential interactions of Cys134 or its mutated counterpart with regions outside of the Forkhead domain cannot be modeled. However, the primary sequence of FOXL2 in this region suggests a disordered chain, which could become ordered upon interaction. Such a potential mechanism might be affected by the mutation, leading to a global altered conformation in the mutant homodimer.

Finally, it has previously been suggested that the DNA-binding specificity of Forkhead factors is partially determined by highly variable sequences in the wings W1 and W2 that could adopt specific conformations and guide the precise positioning of the H3 recognition Helix during DNA binding [38]. Interestingly, these sequences are highly conserved in the case of FOXL2, and the somatic cancer-associated p.Cys134Trp mutation affects the predicted second wing region W2 (Figure 5). This mutation might therefore also disturb the specificity of recognition of a set of promoters relevant to carcinogenesis, or even induce an ectopic modulation of heterologous targets. A potential perturbation of the interaction between mutant FOXL2 and other co-factors, such as Smad3 also remains to be studied.

### Concluding Remarks

Contrary to most BPES-causing mutations, the cancer-associated p.Cys134Trp mutation does not have a strong impact on FOXL2 localization, mobility and transactivation abilities on several reporter promoters. Our luciferase assays indicate that this mutation is neither a dominant-negative nor a loss-of-function mutation on the tested promoters. The case of 3xGRAS-luc suggests that specific signaling pathways could be differentially misregulated by this mutant and would promote malignant transformation while generally maintaining a correct regulation pattern of FOXL2 targets. Understanding the exact pathogenic mechanism underlying the effects of the p.Cys134Trp mutation will therefore require high-throughput unbiased studies, so as to uncover the specifically disturbed FOXL2-regulated functions.

Working with over-expressed FOXL2 variants presents some shortcomings, as some fine-tuned effects, as is often the case for cancer-associated mutations, could be overlooked in this context. However, this also means that studies performed using over-expressed FOXL2 in cultured cells, which will thus be predominant as compared to the endogenous protein, might still yield results informative of the transfected variant, regardless of the cell genotype (i.e. as illustrated by the coherent behavior of WT FOXL2 and the BPES-inducing mutations in KGN cells). It is also worth noting that, as more than 95% of adult OGCTs are carriers of the c.402C>G mutation [12], the relevance of OGCT-derived cell lines not carrying this particular alteration to study this type of cancer is not clear.

## Materials and Methods

### Genomic DNA Extraction and Sequencing

NCI-60 panel genomic DNA were obtained from the National Cancer Institute (after submission of a project involving a formal request) and genomic DNA from 34 CRC cell lines from Sir Walter Bodmer's laboratory [18]. Genomic DNA from cultured KGN (obtained from the Nawata laboratory [16]) and JEG-3 cells (kindly provided by Dr. D. Vaiman, Institut Cochin, Paris) was extracted using a classical Miller's method. The full-length *FOXL2* (*FOXL1*) ORF was amplified using a touch-down PCR approach, using the High-Fidelity Taq Polymerase (Invitrogen). The amplicons were then analyzed using single-pass automated bi-directional Sanger sequencing (BigDye v3.1, Applied Biosystems) and capillary electrophoresis (ABI 3730xl, Applied Biosystems). All primers are available upon request. Sequence analysis was performed manually to detect potential variants. Numbering of sequence variants was performed following the HGVS Mutation Nomenclature Recommendations (http://www.hgvs.org/mutnomen/recs.html), using reference sequence AF301906.1.

### Plasmids

The luciferase reporter plasmids used in this study were described previously: IEX1-luc [39], pFoxL2-luc [40], SIRT1-luc [34], pSODluc-3340 [41], 2xFLRE-luc, 4xFLRE-luc and 4xmFLRE-luc [42], 3xGRAS-luc [29] and OSR2-luc [43].

FOXL2-GFP variants expression vectors are based on a pcDNA3.1 backbone and have been described previously: FOXL2-WT [25], FOXL2-Ala19 [25], FOXL2-I80T [23] and FOXL2-N109K [23]. The FOXL2-C134W expression vector was obtained using junction-PCR as previously described [25].

### Cell Culture, Transfections and Luciferase Assays

COS-7 and HeLa cells, obtained from ATCC, were cultured in DMEM medium, supplemented with 10% FBS and 1% penicillin/streptomycin (Invitrogen-Gibco). KGN cells [16] were grown in DMEM-F12 medium, supplemented with 10% FBS and 1% penicillin/streptomycin (Invitrogen-Gibco). KGN (respectively HeLa) cells were plated 12 h prior to transfection at a density of 4×10^4^ cells.cm^−2^ (2.5×10^4^ cells.cm^−2^) and transfected using the calcium-phosphate method [44]. For luciferase assays, KGN and HeLa cells were seeded in 24-well plates. A Renilla luciferase reporter driven by the RSV promoter (Promega) was included as an internal control of transfection efficiency. Luciferase activity quantification was performed as previously described [25]. Relative luciferase units are the ratio of Firefly over Renilla luciferase activity, and come from at least 5 biologically independent replicates. Statistical significance was estimated by one-way ANOVA or Student t-tests as relevant.

### Exposure of Cultured Cells to Oxidative Stress

To induce oxidative stress, H_2_O_2_ was added to KGN cell culture media to 150 µM for 2 h prior to cell lysis as previously described [34].

### Fluorescence Recovery after Photobleaching (FRAP) Experiments

FRAP experiments were conducted as previously reported [25]. Briefly, COS-7 cells were transfected with either FOXL2-WT-GFP, FOXL2-C134W-GFP or FOXL2-Ala19-GFP in 35 mm plates, perforated, and containing a sterile glass coverslip on the bottom. 48 h after transfection, we performed photobleaching experiments and fluorescent image acquisitions using a LEICA TCS SP2 AOBS confocal microscope. The 30 mW Argon/Neon laser was used at 100% power for photobleaching (2 times, with a lag between bleaching events of 1.635 s). Data were collected from 21 or 22 different cells for each condition. Image analysis was performed with the ImageJ software. Results are mentioned in the text plus or minus SEM.

### In Silico 3D-Modeling of WT and C134W FOXL2 Homodimers

The forkhead domain of FOXL2 was modeled using the crystal structure of the forkhead domain of FoxP2 as template (PDB accession number: 2A07) [26], [37]. The homology module from the Insight 2000 Accelrys software on SGI-O2 was used for the 3D-modeling. The p.Cys134Trp mutant was incorporated in the forkhead domain of FOXL2 using standard rotamer geometry in the biopolymer module of the Accelrys software. The stoechiometry of six Forkhead domains for two DNA molecules which was observed in the experimental FoxP2 forkhead domain crystal structure [37] was used to model the association of the native and mutated FOXL2 forkhead domain to DNA, in order to understand the possible effects of the mutant.

## Supporting Information

Figure S1 Sequencing chromatograms for *FOXL2* in the panels of sequenced cell lines. Portions of the sequencing chromatograms of choriocarcinoma JEG-3 cell line (A) and of the adult ovarian granulosa cell tumor KGN cell line (B) around nucleotide 402 of the coding region. We also report the presence of a new noncoding polymorphism (C) and of already described noncoding SNPs, found in cis (D).
(1.22 MB DOC)

Table S1 Analysis of *FOXL2* genotype in various established cell lines at position c.402.
(0.13 MB DOC)

Table S2 Analysis of *FOXL1* genotype in various established cell lines.
(0.15 MB DOC)
