# Supplementary figures and images for: Functional Exploration of the Adult Ovarian Granulosa Cell Tumor-Associated Somatic FOXL2 Mutation p.Cys134Trp (c.402C>G)
Source: PLoS One. 2010 Jan 20;5(1):e8789. doi: 10.1371/journal.pone.0008789 (PMC2808356; doi:10.1371/journal.pone.0008789)

**FIGURE S1**


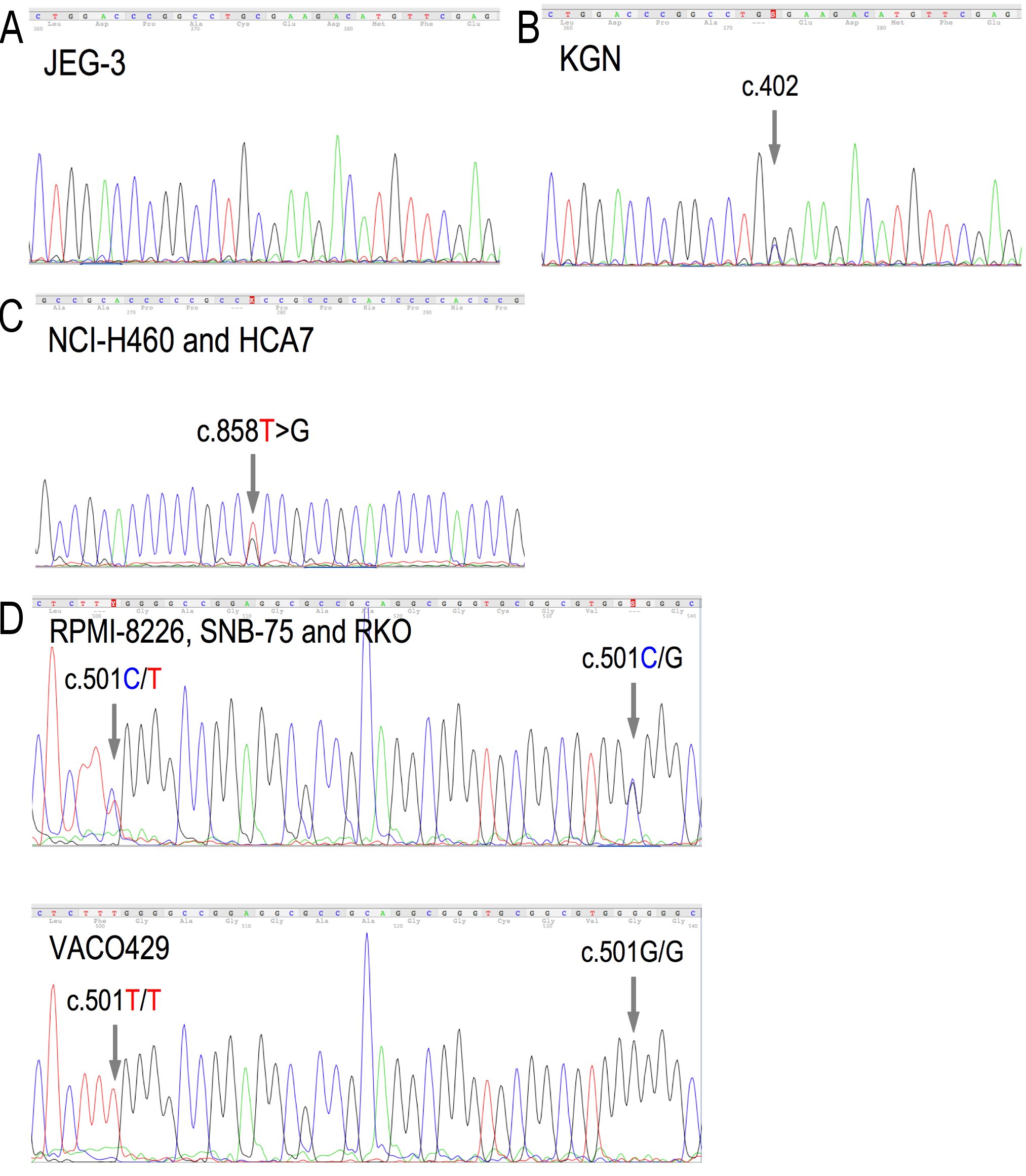

Supplement: Figure S1 — Sequencing chromatograms for FOXL2 in the panels of sequenced cell lines. Portions of the sequencing chromatograms of choriocarcinoma JEG-3 cell line (A) and of the adult ovarian granulosa cell tumor KGN cell line (B) around nucleotide 402 of the coding region. We also report the presence of a new noncoding polymorphism (C) and of already described noncoding SNPs, found in cis (D). (1.22 MB DOC) [file pone.0008789.s001.doc]
