# Supplementary material for: Functional Exploration of the Adult Ovarian Granulosa Cell Tumor-Associated Somatic FOXL2 Mutation p.Cys134Trp (c.402C>G)
Source: PLoS One. 2010 Jan 20;5(1):e8789. doi: 10.1371/journal.pone.0008789 (PMC2808356; doi:10.1371/journal.pone.0008789)
Supplement: Table S1 — Analysis of FOXL2 genotype in various established cell lines at position c.402. (0.13 MB DOC) [file pone.0008789.s002.doc]

**Table S1**

| **Tumor type** | **Cell Line Name** | **Alleles at position FOXL2 c.402** |
| --- | --- | --- |
| **Adult OGCT cell line** | KGN | **C+G** |
|  |  |  |
| **Choriocarcinoma** | JEG3 | C+C |
|  |  |  |
| **Colorectal cancer** | C106 | C+C |
| **Colorectal cancer** | C125-PM | C+C |
| **Colorectal cancer** | C32 | C+C |
| **Colorectal cancer** | C70 | C+C |
| **Colorectal cancer** | C75 | C+C |
| **Colorectal cancer** | C80 | C+C |
| **Colorectal cancer** | C84 | C+C |
| **Colorectal cancer** | C99 | C+C |
| **Colorectal cancer** | CC07 | C+C |
| **Colorectal cancer** | COLO320DM | C+C |
| **Colorectal cancer** | COLO678 | C+C |
| **Colorectal cancer** | HCA46 | C+C |
| **Colorectal cancer** | HCA7 | C+C |
| **Colorectal cancer** | HRA19 | C+C |
| **Colorectal cancer** | LOVO | C+C |
| **Colorectal cancer** | LS123 | C+C |
| **Colorectal cancer** | LS174T | C+C |
| **Colorectal cancer** | LS411 | C+C |
| **Colorectal cancer** | NCI-747 | C+C |
| **Colorectal cancer** | PC/JW | C+C |
| **Colorectal cancer** | RKO | C+C |
| **Colorectal cancer** | SKCO-1 | C+C |
| **Colorectal cancer** | SNU-C2B | C+C |
| **Colorectal cancer** | SW1116 | C+C |
| **Colorectal cancer** | SW1222 | C+C |
| **Colorectal cancer** | SW1417 | C+C |
| **Colorectal cancer** | SW403 | C+C |
| **Colorectal cancer** | SW837 | C+C |
| **Colorectal cancer** | SW948 | C+C |
| **Colorectal cancer** | VACO400 | C+C |
| **Colorectal cancer** | VACO10MS | C+C |
| **Colorectal cancer** | VACO429 | C+C |
| **Colorectal cancer** | VACO4A | C+C |
| **Colorectal cancer** | VACO5 | C+C |
|  |  |  |
| **NCI60 Cell lines** |  |  |
| **Leukemia** | HL-60(TB) | C+C |
| **Leukemia** | K-562 | C+C |
| **Leukemia** | MOLT-4 | C+C |
| **Leukemia** | RPMI-8226 | C+C |
| **Leukemia** | SR | C+C |
| **Non-Small Cell Lung cancer** | A549/ATCC | C+C |
| **Non-Small Cell Lung cancer** | EKVX | C+C |
| **Non-Small Cell Lung cancer** | HOP-92 | C+C |
| **Non-Small Cell Lung cancer** | NCI-H226 | C+C |
| **Non-Small Cell Lung cancer** | NCI-H23 | C+C |
| **Non-Small Cell Lung cancer** | NCI-H322M | C+C |
| **Non-Small Cell Lung cancer** | NCI-H460 | C+C |
| **Non-Small Cell Lung cancer** | NCI-H522 | C+C |
| **Colorectal cancer** | COLO 205 | C+C |
| **Colorectal cancer** | HCC-2998 | C+C |
| **Colorectal cancer** | HCT-15 | C+C |
| **Colorectal cancer** | HT29 | C+C |
| **Colorectal cancer** | KM12 | C+C |
| **Colorectal cancer** | SW-620 | C+C |
| **CNS cancer** | SF-539 | C+C |
| **CNS cancer** | SNB-19 | C+C |
| **CNS cancer** | SNB-75 | C+C |
| **CNS cancer** | U251 | C+C |
| **Melanoma** | LOX IMVI | C+C |
| **Melanoma** | MALME-3M | C+C |
| **Melanoma** | M14 | C+C |
| **Melanoma** | MDA-MB-435 | C+C |
| **Melanoma** | SK-MEL-28 | C+C |
| **Melanoma** | SK-MEL-5 | C+C |
| **Melanoma** | UACC-62 | C+C |
| **Ovarian cancer** | IGR-OV1 | C+C |
| **Ovarian cancer** | OVCAR-3 | C+C |
| **Ovarian cancer** | OVCAR-4 | C+C |
| **Ovarian cancer** | OVCAR-5 | C+C |
| **Ovarian cancer** | OVCAR-8 | C+C |
| **Ovarian cancer** | NCI/ADR-RES | C+C |
| **Ovarian cancer** | SK-OV-3 | C+C |
| **Renal cancer** | 786-0 | C+C |
| **Renal cancer** | A498 | C+C |
| **Renal cancer** | ACHN | C+C |
| **Renal cancer** | CAKI-1 | C+C |
| **Renal cancer** | RXF 393 | C+C |
| **Renal cancer** | SN12C | C+C |
| **Renal cancer** | TK-10 | C+C |
| **Prostate cancer** | PC-3 | C+C |
| **Prostate cancer** | DU-145 | C+C |
| **Breast cancer** | MCF7 | C+C |
| **Breast cancer** | MDA-MB-231/ATCC | C+C |
| **Breast cancer** | HS 578T | C+C |
| **Breast cancer** | BT-549 | C+C |
| **Breast cancer** | T-47D | C+C |
