# Supplementary material for: Functional Exploration of the Adult Ovarian Granulosa Cell Tumor-Associated Somatic FOXL2 Mutation p.Cys134Trp (c.402C>G)
Source: PLoS One. 2010 Jan 20;5(1):e8789. doi: 10.1371/journal.pone.0008789 (PMC2808356; doi:10.1371/journal.pone.0008789)
Supplement: Table S2 — Analysis of FOXL1 genotype in various established cell lines. (0.15 MB DOC) [file pone.0008789.s003.doc]

**Table S2**

| **Tumor Type** | **Cell line Name** | **CDS Variant** | **AA change** | **CDS Variant** | **AA change** | **dbSNP identifier** |
| --- | --- | --- | --- | --- | --- | --- |
| **Breast cancer** | MDA-MB-468 | / |  |  |  |  |
| **Breast cancer** | T-47D | / |  |  |  |  |
| **Breast cancer** | BT-549 | / |  |  |  |  |
| **Breast cancer** | HS 578T | / |  |  |  |  |
| **Breast cancer** | MCF7 | / |  |  |  |  |
| **Breast cancer** | MDA-MB-231/ATCC | / |  |  |  |  |
| **CNS cancer** | SNB-19 | / |  |  |  |  |
| **CNS cancer** | U251 | / |  |  |  |  |
| **CNS cancer** | SF-539 | / |  |  |  |  |
| **CNS cancer** | SNB-75 | / |  |  |  |  |
| **CNS cancer** | SF-268 | / |  |  |  |  |
| **CNS cancer** | SF-295 | / |  |  |  |  |
| **Colon cancer** | COLO 205 | / |  |  |  |  |
| **Colon cancer** | HCC-2998 | / |  |  |  |  |
| **Colon cancer** | HCT-116 | / |  |  |  |  |
| **Colon cancer** | HCT-15 | c.294 G>A | Gly27Ser | c.804 G>C | Ala268Ala |  |
| **Colon cancer** | HT29 | / |  |  |  |  |
| **Colon cancer** | KM12 | / |  |  |  |  |
| **Colon cancer** | SW-620 | / |  |  |  |  |
| **Leukemia** | K-562 | / |  |  |  |  |
| **Leukemia** | MOLT-4 | / |  |  |  |  |
| **Leukemia** | CCRF-CEM | / |  |  |  |  |
| **Leukemia** | RPMI-8226 | / |  |  |  |  |
| **Leukemia** | HL-60(TB) | / |  |  |  |  |
| **Leukemia** | SR | / |  |  |  |  |
| **Melanoma** | MDA-MB-435 | / |  |  |  |  |
| **Melanoma** | LOX IMVI | / |  |  |  |  |
| **Melanoma** | M14 | / |  |  |  |  |
| **Melanoma** | MALME-3M | / |  |  |  |  |
| **Melanoma** | SK-MEL-28 | / |  |  |  |  |
| **Melanoma** | SK-MEL-5 | / |  |  |  |  |
| **Melanoma** | UACC-62 | / |  |  |  |  |
| **Melanoma** | SK-MEL-2 | / |  |  |  |  |
| **Melanoma** | UACC-257 | / |  |  |  |  |
| **Non-Small Cell Lung cancer** | A549/ATCC | / |  |  |  |  |
| **Non-Small Cell Lung cancer** | EKVX | / |  |  |  |  |
| **Non-Small Cell Lung cancer** | HOP-62 | / |  |  |  |  |
| **Non-Small Cell Lung cancer** | HOP-92 | / |  |  |  |  |
| **Non-Small Cell Lung cancer** | NCI-H322M | / |  |  |  |  |
| **Non-Small Cell Lung cancer** | NCI-H226 | / |  |  |  |  |
| **Non-Small Cell Lung cancer** | NCI-H23 | / |  |  |  |  |
| **Non-Small Cell Lung cancer** | NCI-H460 | / |  |  |  |  |
| **Non-Small Cell Lung cancer** | NCI-H522 | / |  |  |  |  |
| **Ovarian cancer** | NCI/ADR-RES | / |  |  |  |  |
| **Ovarian cancer** | OVCAR-3 | / |  |  |  |  |
| **Ovarian cancer** | OVCAR-4 | / |  |  |  |  |
| **Ovarian cancer** | OVCAR-5 | / |  |  |  |  |
| **Ovarian cancer** | OVCAR-8 | / |  |  |  |  |
| **Ovarian cancer** | SK-OV-3 | / |  |  |  |  |
| **Ovarian cancer** | IGR-OV1 | / |  |  |  |  |
| **Prostate cancer** | DU-145 | c.510 G>A | Pro170Pro |  |  |  |
| **Prostate cancer** | PC-3 | / |  |  |  |  |
| **Renal cancer** | ACHN | / |  |  |  |  |
| **Renal cancer** | CAKI-1 | / |  |  |  |  |
| **Renal cancer** | RXF 393 | / |  |  |  |  |
| **Renal cancer** | SN12C | / |  |  |  |  |
| **Renal cancer** | TK-10 | / |  |  |  |  |
| **Renal cancer** | 786-0 | c.814 G>A | Gly272Ser |  |  | rs62051072 |
| **Renal cancer** | A498 | c.814 G>A | Gly272Ser |  |  | rs62051072 |
| **Renal cancer** | UO-31 | / |  |  |  |  |
